# Continued impact of COVID-19 pandemic on clinical and translational science early-career researchers

**DOI:** 10.1017/cts.2022.511

**Published:** 2022-12-07

**Authors:** Colleen Mayowski, Emma A. Meagher, Andrew D. Althouse, Cecilia Patino-Sutton, Maya S. Thakar, Julie L. Welch, Doris M. Rubio, Gretchen E. White

**Affiliations:** 1 Institute for Clinical Research Education, University of Pittsburgh, Schools of the Health Sciences, Pittsburgh, PA, USA; 2 Perelman School of Medicine, University of Pennsylvania, Philadelphia, PA, USA; 3 Center for Clinical Trials and Data Coordination, Department of General Internal Medicine, University of Pittsburgh, School of Medicine, Pittsburgh, PA, USA; 4 Keck School of Medicine, University of Southern California, Los Angeles, CA, USA; 5 Clinical Emergency Medicine, Indiana University, School of Medicine, Indianapolis, IN, USA

**Keywords:** COVID-19, career development

## Abstract

The COVID-19 pandemic had an immediate impact on the lives and work of early-career researchers. We leveraged a cluster-randomized trial and compared survey data collected over two timepoints to explore whether these impacts persisted. Although more than a year had passed, 74% of participants reported that their research was affected in multiple ways in *both* 2020 and 2021. These data suggest that the effects of the pandemic on early-career researchers may be prolonged. Our findings additionally serve as an impetus to identify and implement solutions to early-career challenges that undoubtedly existed before the pandemic, but which COVID-19 brought into the spotlight.

## Introduction

The COVID-19 pandemic impacted early-career biomedical researchers [1–4]. It is important to understand the extent to which the impact has persisted. We have leveraged a cluster-randomized trial that we launched at the onset of the pandemic, the Customized Career Development Platform (CCDP) Trial, to get at this question.

The trial, designed to test the effectiveness of a newly created web-based career development platform for clinical and translational science (CTS) early-career researchers in the USA, included annual online surveys in 2020 and 2021. When COVID-19 arrived in the USA in 2020, we recognized an opportunity to include questions specifically related to the impact of the pandemic that could provide insight into the immediate and longer-term effects by comparing the two timepoints. The objective of this manuscript is to describe the impact of the COVID-19 pandemic on the research productivity of early-career CTS researchers participating in the CCDP Trial and examine the reported change from 2020 to 2021.

## Methods

The CCPD Trial is a cluster-randomized trial of academic institutions that support CTS early-career faculty research scholars (KL2) and pre- and post-doctoral trainees (TL1). We recruited NIH NCATS Clinical and Translational Science Awards (CTSA) funded academic institutions to participate in the CCDP trial. After receiving IRB exempt or “not engaged in human subjects research” designation, TL1 and KL2 program directors at each institution recruited participants for the trial (see Supplemental Fig. 1). To be eligible to participate, all CTS early-career researchers (1) were required to complete an Individual Development Plan (IDP) either by the NIH or their institutional training program and (2) had at least one year left in their training or career development program. Early-career researchers completed anonymous, voluntary online surveys between June and November 2020 and again 1 year later in 2021. Participants were asked to report their age, gender, race and ethnicity, highest degree achieved, and career status. Participants were considered to be underrepresented in sciences if they self-identified as Black, American Indian or Alaskan Native, Native Hawaiian or other Pacific Islander, Hispanic, or from a disadvantaged background [5].

Following the onset of the pandemic, the research team developed and pre-tested specific COVID-19 pandemic impact questions for content, readability, and comprehension. Three questions that related to impact of the pandemic on participants’ research careers and productivity, and their professional/academic life were then added to the originally planned questionnaire. They are described in Supplemental Table 1. Change in the impact of the COVID-19 pandemic on research from 2020 to 2021 was assessed by creating variables that described whether each impact was present or absent in 2020 and/or 2021.

### Data Analysis

This manuscript describes the impact of the COVID-19 pandemic on research productivity of participants in both arms of the trial and excludes participants who were pre-doctoral researchers. We report the number of participants at each participating CTSA hub organized by estimated cumulative incidence of COVID-19 at that geographic location on March 31, 2020 [6]. Descriptive statistics were used to characterize the study population and summarize the change in the impact of the pandemic on research productivity from 2020 to 2021. We tested whether the impact of the COVID-19 pandemic changed from 2020 to 2021 with the McNemar Test. Differences in the impact of the pandemic in 2021 by demographic characteristics (i.e., gender, under-represented in science status, career status, and highest degree) were tested with the Chi-Square test for categorical variables and the Wilcoxon Rank Sum test for continuous variables. We analyzed data using SAS version 9.4 (SAS Institute, Cary, NC, USA). All reported *P*-values are two-tailed; *P* values <0.05 were considered statistically significant. As this was predominantly an exploratory analysis, we did not account for multiple comparisons [7].

## Results

### Participant Characteristics

Three hundred and twelve participants from 27 CTSA sites were included in the analysis for the year 2020 and 175 for the year 2021 (Supplemental Fig. 1 and Supplemental Table 2). Characteristics of participants are shown in Table 1 and were similar between those who responded in 2020 and in 2021. In 2020 and 2021, over half of the participants identified as female (58% and 59%, respectively), White (64% and 63%, respectively), non-Hispanic/Latinx (87% and 88%, respectively), and faculty (57% and 59%, respectively). Eighty-five percent of participants in 2020 and 81% of participants in 2021 reported that their research was affected by the COVID-19 pandemic. The pandemic was reported to have impacted research in 2020 in the following manner: prevented trainees and scholars from completing research as planned (58%), decreased productivity (57%), created competing demands for time (54%), and decreased access to necessary resources (48%). The extent of the reported impact in 2021 was similar: prevented trainees and scholars from completing research as planned (61%), decreased access to necessary resources (50%), created competing demands for time (50%), and decreased productivity (44%). The median number of negative impacts was 3 (25^th^–75^th^ percentile: 1–4) in 2020 and in 2021. The median number of positive impacts was 0 for both timepoints (Table 1).

**Table 1.** Characteristics of participants in the customized career development platform trial at 2020 and 2021

Characteristic	2020		2021	
	*n* = 312	(%)	*n* = 175	(%)
Age (median, 25^th^–75^th^ percentile)	35	(30–38)	35	(30–37)
Gender				
Male	127	(40.8)	68	(38.9)
Female	179	(57.7)	104	(59.4)
Gender minority/prefer not to answer	5	(1.6)	3	(1.7)
Race				
White	197	(64.0)	110	(62.9)
Black	32	(10.4)	14	(8.0)
Asian	48	(15.6)	31	(17.7)
Multi-race	14	(4.6)	12	(6.9)
Other/prefer not to answer	17	(5.6)	8	(4.6)
Hispanic/Latinx ethnicity				
Yes	27	(8.8)	13	(7.4)
No	268	(87.0)	154	(88.0)
Prefer not to answer	13	(4.2)	8	(4.6)
Disadvantaged background				
Yes	66	(21.2)	35	(20.0)
No	239	(76.9)	136	(77.7)
Prefer not to answer	6	(1.9)	4	(2.3)
Underrepresented in sciences				
Yes	99	(33.2)	51	(30.4)
No	199	(66.8)	117	(69.6)
Highest degree				
MD	107	(34.3)	62	(35.4)
PhD	146	(46.8)	84	(48.0)
Other	59	(18.9)	29	(16.6)
Career status				
Post-doctoral fellow	136	(43.6)	72	(41.1)
Faculty	176	(56.4)	103	(58.9)
Research affected by COVID-19 pandemic^a^	266	(85.3)	142	(81.1)
Ways research affected by COVID-19 pandemic				
Prevented me from completing research as planned	183	(58.7)	107	(61.1)
Decreased access to necessary resources	150	(48.1)	88	(50.3)
Competing demands for my time	168	(53.9)	88	(50.3)
Decreased collaboration	83	(26.6)	45	(25.7)
Increased collaboration	31	(9.9)	20	(11.4)
Increased productivity	29	(9.3)	18	(10.3)
Decreased productivity	177	(56.7)	77	(44.0)
Increased funds	18	(5.8)	8	(4.6)
New avenues of research	53	(17.0)	28	(16.0)
Added a COVID-19 research opportunity to my portfolio	69	(22.1)	30	(17.1)
Number of positive ways research affected by the COVID-19 pandemic^b^ (median, 25^th^–75^th^ percentile)	0	(0–1)	0	(0–1)
Number of negative ways research affected by the COVID-19 pandemic^c^ (median, 25^th^–75^th^ percentile)	3	(1–4)	3	(1–4)

a Impact of COVID-19 Survey Questions in Supplemental Table 1.

b Includes increased collaboration, increased productivity, increased funding opportunities, new avenues of research, and added a COVID-19 research opportunity to my research portfolio.

c Includes prevented me from completing research as planned, decreased access to necessary resources and materials, competing demands for my time, decreased collaboration, and decreased productivity.

Change in the impact of the COVID-19 pandemic on research from 2020 to 2021 is shown in Table 2 and it only significantly differed between 2020 and 2021 for decreased productivity such decreased productivity did not change for 71% of participants but 20% of participants stated that the COVID-19 pandemic decreased productivity in 2020 but not 2021 and 10% stated that it did not decrease productivity in 2020 but did in 2021. The impact of COVID-19 pandemic did not significantly differ between 2020 and 2021 for any other measured variable. Three-quarters of participants (75%) said that their research was affected by the COVID-19 pandemic in both 2020 and 2021. Approximately 9% reported that their research was impacted in 2020 but not in 2021, 6% reported that their research was impacted in 2021 but not 2020, and 9% reported that their research was not impacted at either timepoint. Nearly half of participants (47%) reported that the COVID-19 pandemic prevented them from completing their research as planned at both timepoints.

**Table 2.** Change in the impact of COVID-19 on research from 2020 to 2021 for clinical and translational science early-career researchers

	2020/2021 *n* (%)				*P*-value
	Yes/Yes	Yes/No	No/Yes	No/No	
Research affected by COVID-19 pandemic^a^	131 (75.3)	16 (9.2)	11 (6.3)	16 (9.2)	0.34
Ways research affected by COVID-19 pandemic					
Prevented me from completing research as planned	82 (47.1)	17 (9.8)	25 (14.4)	50 (28.7)	0.22
Decreased access to necessary resources	59 (33.9)	26 (14.9)	29 (16.7)	60 (34.5)	0.69
Competing demands for my time	72 (41.4)	27 (15.5)	16 (9.2)	59 (33.9)	0.09
Decreased collaboration	21 (12.1)	25 (14.4)	24 (13.8)	104 (59.8)	0.89
Increased collaboration	4 (2.3)	15 (8.6)	16 (9.2)	139 (79.9)	0.86
Increased productivity	10 (5.8)	5 (2.9)	8 (4.6)	151 (86.8)	0.41
Decreased productivity	60 (34.5)	34 (19.5)	17 (9.8)	63 (36.2)	0.02
Increased funds	4 (2.3)	6 (3.5)	4 (2.3)	160 (92.0)	0.53
New avenues of research	19 (10.9)	11 (6.3)	9 (5.2)	135 (77.6)	0.65
Added a COVID-19 research opportunity to my portfolio	17 (9.8)	23 (13.2)	13 (7.5)	121 (69.5)	0.10

a Impact of COVID-19 Survey Questions in Supplemental Table 1.

The ways in which participants’ research were affected by the COVID-19 pandemic in 2021 significantly differed by career status and highest degree (Table 3) but not by gender or under-represented status (Supplemental Table 3). Significantly higher proportions of faculty members (56% of sample), compared to post-doctoral fellows (44% of sample), reported that the pandemic prevented them from completing research as planned (71% versus 47%, respectively), and that they had competing demands for their time (66% versus 28%, respectively). Faculty members also experienced a significantly higher number of negative impacts than post-doctoral fellows (median: 3 [25^th^–75^th^ percentile: 2–4] versus median: 2 [25^th^–75^th^ percentile: 0–3], respectively). Lower proportions of physician scientists than those with PhDs or other terminal degrees reported that they had decreased collaboration (13% of MDs versus 29% of PhDs and 45% of participants with other terminal degrees), increased productivity (2% of MDs versus 14% of PhDs and 17% of participants with other terminal degrees), decreased productivity (29% of MDs versus 52% of PhDs and 52% of participants with other terminal degrees).

**Table 3.** Impact of COVID-19 on clinical and translational science early-career researchers in 2021, by career status and highest degree

	Postdoctoral Fellow (*n* = 72)		Faculty (*n* = 103)		*P*-value	MD (*n* = 62)		PhD (*n* = 84)		Other (*n* = 29)		*P*-value
	*n*	(%)	*n*	(%)		*n*	(%)	*n*	(%)	*n*	(%)	
Research has been affected by COVID-19 pandemic^a^	54	(75.0)	88	(85.4)	0.08	47	(75.8)	71	(84.5)	24	(82.8)	0.40
Ways research has been affected												
Prevented me from completing research as planned	34	(47.2)	73	(70.9)	0.002	34	(54.8)	53	(63.1)	20	(69.0)	0.38
Decreased access to necessary resources	30	(41.7)	58	(56.3)	0.06	27	(43.6)	47	(52.4)	17	(58.6)	0.35
Competing demands for my time	20	(27.8)	68	(66.0)	<.001	35	(56.5)	39	(46.4)	14	(48.3)	0.47
Decreased collaboration	16	(22.2)	29	(28.2)	0.38	8	(12.9)	24	(28.6)	13	(44.8)	0.004
Increased collaboration	5	(6.9)	15	(14.6)	0.12	7	(11.3)	10	(11.9)	3	(10.3)	0.97
Increased productivity	12	(16.7)	6	(5.8)	0.02	1	(1.6)	12	(14.3)	5	(17.2)	0.02
Decreased productivity	29	(40.3)	48	(46.6)	0.41	18	(29.0)	44	(52.4)	15	(51.7)	0.01
Increased funds	1	(1.4)	7	(6.8)	0.09	1	(1.6)	6	(7.1)	1	(3.5)	0.27
New avenues of research	7	(9.7)	21	(20.4)	0.06	12	(19.4)	9	(10.7)	7	(24.1)	0.16
Added a COVID-19 research opportunity to my portfolio	7	(9.7)	23	(22.3)	0.03	10	(16.1)	11	(13.1)	9	(31.0)	0.08
Number of positive ways research affected by the COVID-19 pandemic^b^ (median, 25^th^–75^th^ percentile)	0	(0–0)	0	(0–1)	0.07	0	(0–1)	0	(0–1)	0	(0–1)	0.53
Number of negative ways research affected by the COVID-19 pandemic^c^ (median, 25^th^–75^th^ percentile)	2	(0–3)	3	(2–4)	<.001	2	(0–3)	3	(1–4)	3	(2–4)	0.10

a Impact of COVID-19 Survey Questions in Supplemental Table 1.

b *P*-value for Wilcoxon Rank Sum test. Includes increased collaboration, increased productivity, increased funding opportunities, new avenues of research, and added a COVID-19 research opportunity to my research portfolio.

c *P*-value for Wilcoxon Rank Sum test. Includes prevented me from completing research as planned, decreased access to necessary resources and materials, competing demands for my time, decreased collaboration, and decreased productivity.

## Discussion

Our initial data collected in June to November 2020 supported earlier findings that the COVID-19 pandemic rapidly impacted early-career biomedical researchers [1, 2, 8, 9]. We wondered whether the CTS early-career researchers participating in the CCDP Trial *continued* to feel the impact of the pandemic on their research productivity in 2021.

When re-surveyed in June to November 2021, we found the impact of the pandemic had indeed persisted. Although more than a year had passed since the COVID-19 pandemic first halted many biomedical research studies, and research enterprises had largely returned, in many ways little had changed. Seventy-five percent of participants reported that their research was affected by the COVID-19 pandemic in *both* 2020 and 2021. Similarly, nearly half of participants (47%) reported that the COVID-19 pandemic prevented them from completing their research as planned at both timepoints. When asked to report specific ways in which their research was affected by the pandemic, there was little difference between timepoints, although the order of affected items changed. We found this to be consistent across our participants, even though they were in varied geographic locations with a varied reported cumulative incidence of COVID-19. These findings contribute to our knowledge of the breadth and magnitude of the COVID-19 pandemic disruption and indicate that the impact of the pandemic may be prolonged.

Interestingly, our exploratory analysis of these data revealed that the ways in which research was affected by the COVID-19 pandemic in 2021 significantly differed by career status and highest degree but not by gender or under-represented status. This is a thought-provoking finding and differs from work by others who have reported that those who identify as female or meet the NIH definition of underrepresentation suffered more negative consequences related to the pandemic [2, 4, 10–12]. This discrepancy creates an opportunity for future research.

We suggest our findings additionally serve as an impetus to identify and implement solutions to early-career challenges that undoubtedly existed before the pandemic and which are now illuminated. Although gathering these solutions is beyond the scope of this article, there is a nascent body of literature deserving of consideration. For example, the work of Cardel, Dean, and Montoya-Williams as well as Davis and colleagues offers a collection of solutions to well-documented barriers facing women investigators [11, 13]. Additionally, Humphries *et al*. examine the unequal pandemic experience of early-career versus established researchers and propose ideas for charting an equitable path forward [10]. This time of increased focus may be our best opportunity to implement long-term solutions that will increase the probability that all have an equal opportunity to achieve career success. We recognize, though, that the results of this study provide no clear path forward. Indeed, because we found differences in impact based on career status and highest degree but not by gender or under-represented status, this likely indicates different interventions may be needed depending upon these characteristics.

Our study has several strengths and limitations. To our knowledge, this is the first study to examine changes in the impact of the COVID-19 pandemic on the research productivity of CTS early-career researchers over time. While this sample is drawn from 27 academic centers, it may not be representative of all CTS early-career researchers. Moreover, only 56% of participants who responded to the COVID-19 questions in 2020 also did so in 2021. However, participant characteristics were similar between the two timepoints and CTSA hubs were geographically diverse. Second, the survey responses measure participants’ perception of impact of the COVID-19 pandemic and are subjective in nature and we did not collect specific examples of how the COVID-19 pandemic impacted them. Future research should expand upon our findings to describe these findings in more detail. Third, we did not collect participants’ field or translational phase of study and participants other commitments, which may be related to research productivity in the face of the pandemic. For example, clinicians faced competing clinical commitments during the pandemic^2^ and basic scientists faced the shutting of their labs while others may have more seamlessly shifted to working from home.

## Conclusions

While the immediate impact of the COVID-19 pandemic on CTS early-career researchers is well-documented, this study provided insights into the sustained impact of the pandemic on the research of early-career clinical and translational scientists. Future studies should consider the long-term consequences of the pandemic and programs should be developed to address them.

## Supplementary material

For supplementary material accompanying this paper visit https://doi.org/10.1017/cts.2022.511. click here to view supplementary material
