# Supplementary material for: Continued impact of COVID-19 pandemic on clinical and translational science early-career researchers
Source: J Clin Transl Sci. 2022 Dec 7;7(1):e7. doi: 10.1017/cts.2022.511 (PMC9879910; doi:10.1017/cts.2022.511)
Supplement: Supplementary file 1 [file S2059866122005118sup001.docx]

**Supplemental Figure 1** – Clinical and Translational Science Award Site and Participant Flow Diagram for the Customized Career Development Platform Trial

Sites approached (k=52)

Sites excluded (k=24)

15 did not respond

5 showed interest, but did not join

1 declined to participate

3 joined, but dropped out before recruitment

Sites recruited (k=28)

Participants (n=351)

1 site excluded – unable to recruit

39 participants excluded

2 at site unable to recruit

9 ineligible because did not require IDP

25 were predoctoral fellows

3 missing COVID data

Have 2020 data

Sites (k=27)

Participants (n=312)

Participants excluded for missing 2021 data

5 withdrew

118 missed assessment

14 missing COVID data

Analysis Sample

175 Have 2021 data

**Supplemental Table 1** – Impact of COVID-19 Survey Questions

| **Question/Prompt** | **Response Option** |
| --- | --- |
| Has your research been affected by the COVID-19 pandemic?^a^ | Yes, no |
| In the past year, has your research been affected by the COVID-19 pandemic?^b^ | Yes, no |
| In which of the following ways has your research been affected by the COVID-19 pandemic? | **Check all that apply**^a^   - Prevented me from completing research as planned - Decreased access to necessary resources and materials - Competing demands for my time - Decreased collaboration - Increased collaboration - Increased productivity - Decreased productivity - Increased funding opportunities - New avenues of research - Added a COVID-19 research opportunity to my research portfolio - Other – please elaborate |
| Positive changes since the COVID-19 pandemic began | **Calculated variable based on variables checked:**   - Increased collaboration - Increased productivity - Increased funding opportunities - New avenues of research - Added a COVID-19 research opportunity to my research portfolio |
| Negative changes since the COVID-19 pandemic began | **Calculated variable based on variables checked:**   - Prevented me from completing research as planned - Decreased access to necessary resources and materials - Competing demands for my time - Decreased collaboration - Decreased productivity |

^a^ Asked in 2020

^b^ Asked in 2021

**Supplemental Table 2**. Number of Participants from Participating CTSA Hubs

|  | n | |
| --- | --- | --- |
| CTSA Hub | 2020 | 2021 |
| Columbia Irving Institute for Clinical and Translational Research | 14 | 7 |
| ConduITS Institute for Translational Sciences at the Icahn School of Medicine at Mount Sinai | 7 | 2 |
| Rockefeller University Center for Clinical and Translational Sciences | 10 | 3 |
| University of Rochester Clinical and Translational Science Institute | 8 | 0 |
| Michigan Institute for Clinical and Health Research | 8 | 4 |
| Clinical and Translational Science Institute at Children’s National: A Partnership with The George Washington University | 5 | 4 |
| Georgetown-Howard Universities Center for Clinical and Translational Science | 15 | 12 |
| Colorado Clinical and Translational Sciences Institute | 8 | 6 |
| Penn State Clinical and Translational Science Institute | 6 | 4 |
| University of Pennsylvania Institute for Translational Medicine and Therapeutics | 8 | 6 |
| Indiana Clinical and Translational Sciences Institute | 22 | 12 |
| University of Florida Clinical and Translational Science Institute | 17 | 9 |
| Utah Clinical and Translational Science Institute | 16 | 8 |
| Johns Hopkins Institute for Clinical & Translational Research | 8 | 7 |
| UW Institute for Clinical and Translational Research | 12 | 9 |
| Washington University Institute of Clinical and Translational Sciences | 23 | 11 |
| Scripps Research Translational Institute | 12 | 2 |
| Southern California Clinical and Translational Science Institute | 12 | 7 |
| UC Davis Health Clinical and Translational Science Center | 14 | 10 |
| UC San Diego Altman Clinical and Translational Research Institute | 5 | 2 |
| UCLA Clinical and Translational Science Institute | 15 | 8 |
| UCSF Clinical and Translational Science Institute | 16 | 15 |
| University of Alabama at Birmingham Center for Clinical and Translational Science | 7 | 2 |
| Oregon Clinical and Translational Research Institute | 12 | 5 |
| Duke Clinical and Translational Science Institute | 11 | 6 |
| North Carolina Translational and Clinical Sciences Institute (NC TraCS) | 13 | 7 |
| University of Kentucky Center for Clinical and Translational Science | 8 | 7 |

CTSA hubs organized based on estimated cumulative incidence of COVID-19 at that geographic location on March 31, 2020 (<https://www.cdc.gov/mmwr/volumes/69/wr/mm6915e4.htm>). Shading indicates quartile of COVID-19 cumulative incidence with dark gray indicating the highest cumulative incidence of COVID-19 on March 31, 2020.

**Supplemental Table 3** – Impact of COVID-19 on clinical and translational science early-career researchers in 2021, by gender and underrepresented status

|  | Male  (n=68) | | Female  (n=104) | |  | Underrepresented (n=51) | | Not Underrepresented (n=117) | |  |
| --- | --- | --- | --- | --- | --- | --- | --- | --- | --- | --- |
|  | n | (%) | n | (%) | P-value | n | (%) | n | (%) | P-value |
| Research has been affected by COVID-19 pandemic^a^ | 55 | (80.9) | 86 | (82.7) | 0.76 | 43 | (84.3) | 94 | (80.3) | 0.54 |
| Ways research has been affected |  |  |  |  |  |  |  |  |  |  |
| Prevented me from completing research as planned | 43 | (63.2) | 63 | (60.6) | 0.73 | 32 | (62.8) | 70 | (59.8) | 0.72 |
| Decreased access to necessary resources | 33 | (48.5) | 55 | (52.9) | 0.58 | 28 | (54.9) | 57 | (48.7) | 0.46 |
| Competing demands for my time | 35 | (51.5) | 52 | (50.0) | 0.85 | 26 | (51.0) | 60 | (51.3) | 0.97 |
| Decreased collaboration | 15 | (22.1) | 30 | (28.9) | 0.32 | 12 | (23.5) | 30 | (25.6) | 0.77 |
| Increased collaboration | 6 | (8.8) | 14 | (13.5) | 0.35 | 5 | (9.8) | 15 | (12.8) | 0.58 |
| Increased productivity | 4 | (5.9) | 14 | (13.5) | 0.11 | 8 | (15.7) | 9 | (7.7) | 0.11 |
| Decreased productivity | 31 | (45.6) | 45 | (43.3) | 0.76 | 25 | (49.0) | 48 | (41.0) | 0.34 |
| Increased funds | 2 | (2.9) | 6 | (5.8) | 0.39 | 2 | (3.9) | 6 | (5.1) | 0.74 |
| New avenues of research | 11 | (16.2) | 17 | (16.2) | 0.98 | 6 | (11.8) | 20 | (17.1) | 0.38 |
| Added a COVID-19 research opportunity to my portfolio | 9 | (13.2) | 21 | (20.2) | 0.24 | 6 | (11.8) | 22 | (18.8) | 0.26 |
| Number of positive ways research affected by the COVID-19 pandemic^b^ (median, 25^th^-75^th^ percentile) | 0 | (0-1) | 0 | (0-1) | 0.33 | 0 | (0-1) | 0 | (0-1) | 0.98 |
| Number of negative ways research affected by the COVID-19 pandemic^c^ (median, 25^th^-75^th^ percentile) | 3 | (1-4) | 3 | (1-4) | 0.90 | 3 | (1-4) | 2 | (1-4) | 0.63 |

^a^ Impact of COVID-19 Survey Questions in Supplemental Table 1.

^b^ P-value for Wilcoxon Rank Sum test. Includes increased collaboration, increased productivity, increased funding opportunities, new avenues of research, and added a COVID-19 research opportunity to my research portfolio.

^c^ P-value for Wilcoxon Rank Sum test. Includes prevented me from completing research as planned, decreased access to necessary resources and materials, competing demands for my time, decreased collaboration, and decreased productivity.
